# Roper romps, feeling rules, and the “appropriately inappropriate” management of joy

**DOI:** 10.3389/fsoc.2026.1738791

**Published:** 2026-04-22

**Authors:** Jessica S. Pearce

**Affiliations:** Department of Sociology, Anthropology, and Human Development & Family Science, University of Louisiana at Lafayette, Lafayette, LA, United States

**Keywords:** emotion management, emotion work, feeling rules, gender, joy, roper romps

## Abstract

The author explores the management of emotion at a recent pop culture leisure activity, Roper Romps. What emotions are expressed at local Roper Romps? How does the news reporting about these events assist in the construction of feeling rules? How does this situational context affect emotion management? To answer these questions, the author conducted a thematic analysis of local and national news stories reporting on Roper Romps between 2023 and 2024. The most frequent emotion observed was joy, which intersected with other emotions. Furthermore, though limited, results demonstrate the continued relevance of Hochschild’ work (1979, 1983), the significance of feeling rules and emotion management techniques at a unique pop culture event. Pop culture trends, especially those that expect women to be “appropriately inappropriate” are opportunities for women to emotionally manage the routine expectations of everyday life.

## Introduction

The author seeks to contribute to work on sociological literature, primarily, through the application of Hochschild’s (1979, 1983) feeling rules and emotion management. There are various techniques by which we suppress feelings they do not want to feel and to evoke those we want to feel (Hochschild, 1979). These proscriptive ideas are often dictated by what we are told, shown, or what we have previously experienced (Hochschild, 1983). Literature also has established that these rules are regularly unequally applied, based on race, gender, or sexuality (Wingfield, 2010; Hochschild, 1983; Mai, 2022). Finally, social gatherings are an apt environment for studying emotion as they can coalesce and transform participants. When people come together to engage in similar behaviors, focused on the same goal, feelings can become intertwined and heightened (Durkheim, 1912/1995). Emotion norms are connected to situational context; different situations require different feelings (Hochschild, 1979). Normative expectations are constructed across all contexts, including those which emerge within pop culture (Harrington and Bielby, 2018), indicating to consumers what identities are and are not appropriate.

Thus, the author poses several questions. What feeling rules are constructed at Roper Romps and how does the news media contribute to this construction? How do these feeling rules potentially allow women to tap into pop culture phenomena to express joy? What evidence of emotion management is there in this process? I contend that Roper Romps (large gatherings of citizens who dress as Mrs. Roper or other Three’s Company characters) leverage a pop culture phenomenon to create a space for women to elicit appropriate emotions in inappropriate ways, expanding the sociology of emotions literature to its application to unique pop culture events.

Using Hochschild’s theory of emotion work, this study argues that the news media contribute to cultural definitions of fun and joy, often reinforcing existing emotion norms. Roper Romps offer opportunities for women, people of color, and LGBTQ participants to craft alternate identities and renegotiate what leisure means. Leisure is not only about “free time” but acts as a site for collective emotional expression and the transformation and modification of cultural expectations, especially for those groups who are disproportionately affected within society more broadly. By defining the feeling rules, the news media reveals how power operates through everyday activities and how it can be contested and transformed for the individuals who are subjected to these emotional standards. The mediating role of journalistic sources is a legitimizing force in the context of emotion and community (and self) expression.

### Roper romps

First airing in 1976, *Three’s Company* began as a sitcom about three tenants, Jack, Janet, and Chrissy. In the series’s opening premise, the three characters claimed Jack was gay to legitimize their co-ed living situation in the eyes of the apartment’s owners, the Ropers. The audience was invited to laugh at the characters’ circumstances and at their efforts to maintain their ruse. As hijinks ensued, the audience got to know not just the tenants but also the Ropers, Stanley and Helen. Helen’s character, in particular, stood out;

Jack, Janet, and Chrissy, could not have asked for a kinder landlady. Helen Roper is a warm, good-hearted woman who loved to laugh and be affectionate with her husband. If only Stanley wasn’t such a stick in the mud… She is known for her kooky kaftans, bright colored jewelry and wiry, bright red hair (DLT Entertainment Ltd, 2025 para. 1)

Fifty years later, Mrs. Roper has re-emerged as a cultural icon across the United States and has become the central figure in a new leisure event—the Roper Romp.

Roper Romps originated in New Orleans at the Southern Decadence parade in 2013; that year, fifty attendees dressed as Mrs. Roper as part of the LGBTQ-centered celebration. Bud Moore, the creator of the Roper Romp and the first Roper Romp Facebook page chose a costume in which parade-goers “can dress how they want, but it’s a recognizable character” (Coviello, 2023). His decision kickstarted a movement that has snowballed, becoming larger and more popular just in the last 2 years. Roper Romps have invited participants to channel Mrs. Roper’s bawdy persona and relish the experience of fun and silliness for a day. Dozens of Roper Romps have been held over the last several years, with attendance sometimes numbering in the thousands. The forms of events have varied, although they were often centered around a pub crawl or tour of local businesses. Organizers have incorporated other activities such as trivia, best-dressed competitions, karaoke, games of limbo, and charity drives. However, scholars have yet to explore the potential significance of Roper Romps and how they have utilized emotion management.

Though the show is a sentimental classic for many, some have argued that the *Three Company*’s plotlines are no longer reflective of current cultural beliefs. Jack’s attempts to use homosexuality as a front to justify living with two attractive female tenants has aged poorly, leveraging overt stereotypes and homophobic jokes for laughs (Pagnotti, 2024). Mr. Roper’s conventional views on heterosexual roommates also propped up many problematic storylines. In contrast, Mrs. Roper, who had focused on expressing her own sexual desires toward her (uninterested) husband, was aware of the farce and often colluded with her tenants to fool her spouse. Her character exuded confidence, sexuality, and open-mindedness. The landlady’s personification of progressive values continues to align with modern views of gender and sexuality, appealing to many.

### Feeling rules and emotion management

Public gatherings, like Roper Romps, are sites for emotional expression. The author has grounded this analysis in Hochschild’s (1979, 1983) concept of feeling rules and emotion management. Feeling rules dictate emotions appropriate to situations (Hochschild, 1979, 1983); and social customs offer clear examples of such rules. “Sets of feeling rules contend for a place in people’s minds as a governing standard with which to compare the actual lived experience” (Hochschild, 1979, p. 567). Thus, people regularly enter situation in which they expect to feel a specific emotion. Furthermore, various situational contexts can each demand individual emotional norms. However, feeling rules do not automatically inspire customary emotions. Emotion management, then, is a process that guides feelings (Hochschild, 1979, 1983). Though feeling rules may be in play, individuals may find expected emotions to be absent. Using emotion management, inappropriate emotions can be stifled or transformed, and appropriate feelings can be elicited. Management through surface acting, like smiling, expresses the desired emotion, even if the emotion remains unfelt (Hochschild, 1979, 1983). Deep acting, in contrast, directs how individuals actually feel, and requires individuals to work harder to evoke wanted feelings (Hochschild, 1979, 1983). One way to accomplish this is through cognitive deep acting. By controlling their thoughts, people can also generate desired feelings. Medical students have been found to “use the patient” to distract from feelings of discomfort by evoking blame (Smith and Kleinman, 1989). Emotions can also be suppressed by deep acting, justifying, or excusing personal behavior to mitigate feelings of guilt and remorse (Scott and Lyman, 1968). Furthermore, individuals can engage the body in deep acting, focusing on the somatic experience of emotion. Johnston (2021) has discovered the utility of spiritual practices in the bodily management of emotion. Her analysis included observation of a yoga community, during which she uncovered the use of pranayama (breathwork). One yoga instructor described this technique: “When you are angry at someone or frightened or anxious, you can use the prana (breath) to work on that to saturate it, let it be clear, and release it” (Johnston, 2021, p. 591). Similarly, Mai (2022) has found that Vietnamese LGBTQ activists could perform cognitive and bodily forms of emotion work to generate the needed emotions both on front stage and backstage. Finally, Hochschild (1979, 1983) has outlined the process of expressive emotion work. By honing outward emotional expressions, individuals may extract the expected feeling; however, sometimes individuals need others to assist them in experiencing a specific emotion. Thoits (1996) has documented the power of expression work in her analysis of a “psychodrama-based encounter group” where members engaged in enactments to provoke extreme emotional responses. Though ‘extreme” emotion is not always needed; simple acts, such as smiling at someone or giving them a physical reminder can also be used in this process (Grandey, 2000).

Socialization undergirds much of our understanding of emotion and its appropriateness (Hochschild, 1979). However, Goffman’s (1959) dramaturgical theory is also relevant to emotion in terms of performance. The definition of the situation lays a foundation for the construction of appropriate emotions. The context of a situation establishes a backstage and front stage area on which people rest and prepare for their performance and perform their roles, respectively. Whatever the persona dictated by the situation, people are expected to play it, while making it as authentic as possible. Like Hochschild, under the umbrella of dramaturgy, emotions are present and people into the performance. If the actors, step outside the bounds of their character, then others can institute aligns actions (Stokes and Hewitt, 1976) to remind the performers to stay “in character.” Expressing the correct emotions assists in the credibility and continued progression of the situation.

### Gender and emotion

Situations can affect people’s experiences in different ways. Feeling rules are often constructed unequally across a spectrum of identities. Indeed, Hochschild’s (1983) work on emotion management and feeling rules is based on her ethnographic work on Delta airlines. She outlines the ways in which flight attendants and bill collectors were expected to elicit feelings, though on distinct ends of the emotional continuum (Hochschild, 1983); however, scholars have also examined feeling rules and emotion management in ways unrelated to paid work. Gender is a common identity by which people characterize and separate feeling rules. Studies on abortion (Keys, 2010), foodwork (Muñoz and Quirke, 2022), prostate cancer (Andreasson et al., 2023), and hair loss (Olson, 2024) have offered experiential insights into the process of emotion work and its connection to social identity. Women have often used emotion management techniques to coordinate their emotions with the proper feeling rules. Again, Hochschild’s (1983) book explored flight attendants and how they, as women, were asked to display emotions that aligned with stereotypical female feelings or care, concern, and happiness. These feeling rules were designed to make the (male) passengers feel “at home.”

Race also defines rules of feeling. In her study of black professional. Wingfield (2010) reveals that in the workplace, there are emotions that are for “whites only.” Emotions like anger are considered appropriate in some professional contexts; however, for Black workers anger (even if justified) is ostensibly off-limits because of the racial stereotypes it evokes. Workers do not want to take the chance that their feelings will be misinterpreted and used against them because of their race. The intersection of race and gender also dictate the feelings that can and cannot be expressed (Cottingham et al., 2017) found that black female nurses in a predominantly white workplace, must do work specific to these conditions to manage emotions that results from racially charged incidents.

Feeling rules are also relevant to the vast experiences of sexual identity, Mai (2022) found that Vietnamese LGBTQ activists could perform cognitive and bodily forms of emotion work to generate the needed emotions both on front stage and backstage. The kind of emotion work in which they engaged was influenced by the positive feeling rules affecting the front stage work of activism. In Brummer’s (2024) examination of students at the intersection of citizenship, emotions, and place, she found that the classroom as a constructed situation has its own feeling rules, offering for some an emotionally safe environment, compared to other unsafe spaces or situations.

### Leisure and emotion

The author also looked outside of sociological literature and has included work from the area of leisure studies. Romps are recreational activities, and this body of scholarship may illuminate some of the emotional mechanisms at play. For instance, “grandtravel,” broadly conceptualized as children traveling with their grandparents, has provided a temporal experience in which family members co-create emotion (Gram et al., 2019); by traveling together, grandchildren and grandparents use emotion to enhance their wellbeing. Similarly, in their research on running groups, Desjardins and Ketterling (2023) have found that runners not only use physical activity as an outlet for difficult or troublesome feelings, but also to produce positive feelings. In her study of Australian women who struggled with depression, Fullagar (2008) has found that the “emotion play of leisure” allowed women an outlet to resist and recover from the negative emotion work of their everyday lives. Women acknowledged that their social connections played a significant role in this process (Fullagar, 2008). Most recently, Hybholt and Spotswood (2025) have found that for middle-class Danish women, lifelong regular physical activity (LRPA) allowed them to “switch off” negative feelings and “switch on” positive ones. Thus, leisure activities gave them a mechanism to manage their emotions. Leisure activities can offer utility for those who need to feel positive emotions. Traveling and exercise can produce warm feelings, especially when these activities are shared with others. Though the authors have not framed this process as emotion management, it fits Hochschild’s (1979) description of this notion. Thus, engaging in such pursuits can allow participants to intentionally suppress or elicit the feelings they desire.

Cosplay is another form of leisure through which we can examine emotions, management, and feeling rules. Cosplay is a portmanteau of “costume play;” cosplayers dress in costume and act as characters for fun usually in the presence of others cosplayers. It is a unique subculture (Lamerichs, 2014) that offers an opportunity for play (Lamerichs, 2011, 2014), and the opportunity to generate fun and joy. Ibarra and Petriglieri (2010) have examined the use of identity play within organizational structures, delineating how people test potential future identities through cosplay. Even though cosplayers may not really want to become fictional characters, by dressing in costume they can channel certain aspects of themselves that they want to be. This includes the feelings of positive emotions.

Like deep acting, the body is also a critical element in the creation of emotion in cosplay. In their examination of cosplayers, Masi de Casanova et al. (2021, p. 813) have found that the act of embodiment can elicit “pleasurable feelings of empowerment or liberation”; participants noted that they did not feel that they became the character they played, but instead, cosplay offered an escape from their daily lives and the opportunity to experiment. Furthermore, Masi de Casanova et al. (2021, p. 813) have found that part of the joy cosplayers experienced came from the complexity of acting in character; “[i]t is not quite method acting or spirit possession, but there is some loss of control over the body that leads to pleasurable immersion.” Using dramaturgical theory to understand the spaces which cosplayers occupy provide the foundation for their study and has exposed the “blurred boundaries” that exist between front stage and backstage (Masi de Casanova et al., 2021; Goffman, 1959), but even the preparation of their appearances leads cosplayer to bonding (Hodkinson, 2002). Dressing up as a character can lead to positive feelings in another way, including through physical and cognitive “escape.” Even if the character is fictional, is does not have to diminish the power of embodiment and, thus joy and escape.

It is not only *what* you are doing when you are having fun that may be important, but also *where* and with *whom*. Importantly, fun is sociological, shaped by the structural opportunities situated in time and space (Fine and Corte, 2017). The physical spaces and social environment affect how we experience fun. Once strong feelings are produced, participants can generate “collective effervescence” (Durkheim, 1912/1995). Further, Fine and Corte (2017) have described the theoretical aspects of group fun. As participants share intense communal emotions, they can create and reinforce a feeling of cohesion (Collins, 2004). Indeed, fun assists in the maintenance of the interaction order and its coordinating events (Mead, 1934); collective action includes shared emotion, which can further act as a “commitment mechanism” (Simmel, 1984). The emotional experience of fun in the present continues and connects people after the fact (Goffman, 1981) leading to the discovery of an imagined community (Wettergren, 2009). The presence of others is a powerful force, and can contribute to the emotions one experiences, or wants to experience, like religious ceremonies. Gender, costumes, the shared presence of others and, who and where we are gathering, are key elements in the construction of feeling rules and the expression and management of emotions.

Finally, popular culture offers some insight to seemingly retro phenomenon. Nostalgic entertainment, like older television shows and their characters can elicit joy from their connections to the past (Reynolds, 2011). Similarly, Askin and Mauskapf (2017) in their analysis of Billboard’s Hot 100 charts, argue that the popularity of a song is connected to its similarity to existing songs. Songs that moved up the charts were those that were new but not significantly different, they reminded the listener of songs they had heard before. Furthermore, Reynolds (2011) claims that pop music is submerged in the past; many of the bands and songs that were popular decades ago heavily influence what music is popular today. Though focused on music, he extends his argument to other areas of popular culture as well—television, movies, theater, and fashion among others. Pop culture is fun and offers fans an avenue to environment where they can and should pursue joy.

## Methods and materials

The aim of the data collection process was to obtain access to news stories between 2023 and 2024, which were pulled from several sources. First, the author searched the University library’s online databases, NewsBank and Nexis Uni, for news articles on Roper Romps. Second, to ensure a more robust sample, the author explored Google News using a standard internet browser. Some articles were duplicated across two or more of these sites; however, it was counted only once. Searches resulted in 114 sources. Two articles were indirect or inaccessible; one was irrelevant and one was hidden behind a paywall and was inaccessible. An advantage of using existing new stories is that there were no concerns about human subjects as the data were public news stories.

Once the data were collected, the author conducted a thematic analysis of the written content, searching for relevant patterns related to emotion. Using Braun and Clarke’s (2006) method, the author read through the articles to familiarize herself with the existing material, taking note of potentially relevant mentions and descriptions of emotion. Utilizing initial thematic codes, the author next developed sensitizing concepts as buckets of meaning for continued analysis. Concepts were pulled from previous studies on feeling work (Hochschild, 1979, 1983), emotion work (e.g., Thoits, 1996) and leisure studies (e.g., Desjardins and Ketterling, 2023; Yamashita et al., 2019). Patterns coalesced around joy, community, and freedom, and how participation in a Roper Romp affected their emotional state. The author also observed photographs and recorded video broadcasts among the dataset. News stories and video transcripts offer an opportunity to examine emotional expressions as both organizers and attendees described their experiences and personal feelings within the relevant social context. In contrast, photographs were limited to analysis of expression. Thus, the activities, location, and perceived emotions captured at the Roper Romps can be described as they are depicted, but the social processes grounding the events cannot be explored. In the final phase, the author re-examined the data to confirm the constructed themes, which are discussed in the following section.

The author chose existing news stories as the source of data for several reasons. The author sensed that the overarching intention among the more robust news stories about Roper Romps was exploratory and descriptive, dovetailing with the author’s intentions. Given that Roper Romps are a novel phenomenon, the reporters detailed these events and the participants’ interest in participating. Though the author is unable to concretely determine intention from existing data, and though the findings from these data may be limited (Neuman, 2014), this does not preclude its use.

Practically, secondary data offers a valid starting point for exploration of a topic. The daily reality of academics composed of a mix of service, teaching, and research duties often constrains time for research projects (Dufour and Richard, 2019). Secondary data are available and allow academics to engage in a fruitful research agenda while balancing a busy schedule. Furthermore, journalists, especially those in smaller communities, are familiar with the local community and vice versa, granting reporters access in a way that an outside researcher would not receive. Though the research questions for existing data may lack a positivist stance, the scholarly relevance of a corpus of text should not be dismissed because it is accessible (Hofferth, 2005). Quantitative analysis of secondary data is common, if not routine for many research areas, yet the use of qualitative analysis is often questioned (Dufour and Richard, 2019) even though advancements in qualitative methodological processes continue (Cheong et al., 2023).

For this project, existing news stories afford the author a chance to examine the portrayal of Roper Romps in the media and to gain an exploratory glimpse into the participants’ experiences and the sociological relevance of such events. The use of publicly available data like news stories provided a method to expand the geographic scope of data for low-cost preliminary purposes (Cheong et al., 2023).

Journalistic sources also offer scholars a new perspective on the creation of feeling rules. Feeling rules or the groundwork for normative and feeling rules are found in all sources produced by humans. Reading about past accounts from participants in a unique event provides a unique method with which to track these rules. Emotions can be transmitted through media and can influence not only how we think about the world, but also what we feel. Recent qualitative research on news and emotion includes Olson et al.’s (2025) examination of qualitative climate change and climate anxiety and a qualitative comparative analysis of protest movement coverage and sentiment (Amenta et al., 2024).

News media are sites for the collective construction and public dissemination of emotion norms. Readers encounter ideas and events they may not directly experience. The stories, headlines, photographs that they observe frame and normalize emotional responses to specific events. The study of news media allows us to monitor how a community constructs emotions, how these emotions are attached to identities and signal which feelings are allowed for various groups and when they are allowed to express them. By acting as a mediator between accounts and readers, journalistic sources offer a legitimate, community-oriented lens through which to view events. Thus, such accounts become the normative standard for promoted events, yet the subsequent collective experiences allow for the transformation or adaptation of the required feeling rules.

Many scholars have successfully used the analytical method developed by Braun and Clarke (2006). For example, Ringenberg et al. (2024) have studied the online chats between law enforcement officers and offenders and offenders and underage victims. As part of a mixed methods study, Tint et al. (2025) have observed the top Instagram posts for #TraumaticBrainInjury. Finally, Akinlotan and Jalo (2024) have examined published qualitative literature about the experiences of older adults during the COVID pandemic. A key advantage of Braun and Clarke’s (2006) method is the flexibility allowed for qualitative researchers in the development of analytic themes.

## Results

The author identified several analytical axes, connecting the data and scholarly literature. These topics include leisure, embodiment, and identity play, gender and the social construction of fun, and news stories and the dissemination of feeling rules. These themes reveal the nuance that exists within a specific context for collective leisure. Feeling rules are socially constructed through collective action, shared with others, and are mediated through the lens of news accounts, which transform these emotional cues into normative standards of collective emotion.

### Leisure, embodiment, and identity play

A key element of Roper Romps is dress and appearance. Participants used their bodies to channel emotions and explore their emotions by temporarily accepting a fictional identity. Deep acting (Johnston, 2021), features of cosplay (Masi de Casanova et al., 2021), and identity play allow participants to test possible selves (Ibarra and Petriglieri, 2010) in a fun and protected social environment. Roper Romps offer an opportunity to observe how leisure practices serve as intentional mechanism for mood regulation for participants.

The fictional character of Mrs. Roper was one that demanded attention. “She is known for her kooky kaftans, bright colored jewelry and wiry, bright red hair” (DLT Entertainment Ltd, 2025 para. 1). Thus, like cosplayers, the costume for Mrs. Roper was a foundational component in creating the Roper Romps. By wearing the caftan, the wig, and other accessories participants indicated to others that they accepted the part they were expected to play and the personality and emotions incumbent within. Through the accounts of past Rompers, potential participants understood the expectations to which they agreed. As described by Rompers, the possible self-consciousness was an avenue to happiness. They were being asked to become slightly uncomfortable by becoming a fictional character, through which they could potentially *become* joyful. “It was a time to sit back and watch everybody just having a ball, having such a good time—no agenda. People would come, and you just laughed at what they looked like in these goofy wigs” (Article 40). Like in cosplay (Lamerichs, 2014) the costume, being in character, elcitied positive emotions; this was part of managing their emotions to align with the required normative and feeling rules.

Many Rompers remembered Mrs. Roper from their childhood or young adulthood. Like Askin and Mauskapf’s (2017) study of popular music, Mrs. Roper’s appeal is an example of a fashionable pop culture trend (Romps) remind participants of the past. Roper Romps allowed them to tap into memories of their youth, eliciting feelings of joy mixed with nostalgia.

In a world where the past often feels like a foreign country, the Mrs. Roper Romp serves as a delightful bridge back to a time of simpler pleasures, laugh tracks, and the kind of community spirit that can only be found when people come together to celebrate something they love (Article 27).

Potential participants were reassured that if they attended, they could revisit their younger years and be reminded of things they loved. Lee Harrington and Bielby (2010, p. 445) “suggest that as normative adult life destabilizes, cultural objects are increasingly providing a reference point for navigating the trajectory through adulthood and later life.” Roper Romps and Mrs. Roper’s character are positioned as a touchstone for attendees, especially for those who are middle-aged or older. The messages from past accounts indicated that familiarity with Mrs. Roper would help Romps to get into character; this is much like the backstage area where cosplayers dress and prepare for their events (Masi de Casanova et al., 2021; Goffman, 1959). The character’s appeal was also attributed to participants’ ability to identify with Mrs. Roper in a way they could not when they were young. The data revealed an appreciation for her perceived authenticity and her ability to stand out. One participant shared her perspective on Mrs. Roper:

[S]he wanted to be like Chrissy, played by Suzanne Somers, and saw Roper as ‘some kooky old lady with funny clothes.’ Looking back years later, she realizes she is more similar to Roper. ‘She just puts it all out there, and now that I’m 50 I can appreciate her so much more’ (Article 109).

There are hints at a kind of cyclical fandom (Hills, 2005); even if participants were not “serious” fans, their awareness of Mrs. Roper makes her character an easy outlet for fun. For those who are familiar with the show, Rompers were asked to tap into her persona to find some sense of solidarity and inspiration and potentially use this as a mechanism to feel joy.

However, many past participants were unaware of Mrs. Roper and where she originated, or they were delighted by other people’s ignorance of the character and the event generally. Articles describe how non-participants derive pleasure from seeing people dressed as Mrs. Roper. Several participants across the Romps described their run-ins with non-participants; bystanders were confused by the group, who they were, and what they were doing. “‘What’s going on here?’ Matthews asked me. ‘It’s amazing!’ Once I explained, she said: ‘Where can I sign up for it next year?’” (Article 32). Reporters and participants often connected this discrepancy to generational gaps. Passersby often mistook them for other well-known red-headed personalities, real and fictional. Most noted is Little Orphan Annie from the eponymous comic strip: “At first glimpse, you would think they had gone as middle-aged Annies, but nope, they were dressed as Mrs. Roper” (Article 3). Ice Spice, an American rapper, and Ms. Frizzle from *The Magic School Bus*, an animated children’s television series were also mentioned. “As one passerby noted, ‘I’ve seen the Naked Bike Ride, but I’ve never seen anything like this’” (Article 32). These instances also tap into the relative deviance created by Roper Romps. People may be confused by what’s going on, but ultimately, they come to view these events as appropriately inappropriate. They violate folkways, but it is partly through violation that participants derive their pleasure, fulfilling the feeling rules established by Roper Romps.

In many cases, the caftan and the accessories imitating Mrs. Roper were required, expecting participants to commit fully to Mrs. Roper’s persona. However, for many Romps, the rules on costuming were more inclusive. Anyone could dress as any character, if it was a character from *Three’s Company*. “Participants can come dressed as other characters from the show, someone from the 1970s, or as themselves. ‘Helen is a spirit,’ she said. ‘It’s just doing your own thing and having fun’” (Article 15). Even if the other characters were dissimilar to Mrs. Roper’s personality, their presence on the show was an allowable exception to the Romp experience. For others the (full) costume was unnecessary. Instead, the focus was on embodying the spirit of Mrs. Roper through participation, not just dressing like her. This included people of various genders and sexualities. Again, the feeling rules of emotion, instruct participants to feel joy even if they do not fully “accept” the concomitant rules for costuming. “Helen’s equal opportunity muumuu now encompasses all genders, ages, races, and sexualities” (Article 76). Like the dress for women, men, and people of various sexualities could act appropriately inappropriate by following the rules of feeling. Even here the costumes or proximity to costumed participants function as a tool to both follow the rules and elicit desired emotion. People should not avoid attending because they do not fit the stereotype of what Mrs. Roper is “supposed to look like.”

The romp with Mrs. Roper provided participants with an opportunity to forget about work, parenthood and stress in general by spending time dressing up as a character from a popular show they grew up watching on TV. ‘I think everybody,’ Durkin said, ‘just kind of needed something like that.’ Participants can “let go” and relax and have fun. ‘Honestly, if I were a debutante, this would’ve been my coming out party,’ Lepere said with a big smile. ‘Life is pretty tough in general, and to be able just to escape and have fun was incredible. This is a judgment-free zone. Nobody is thinking anything except that we’re all Helens’ (Article 76).

Another normative expectation attached to Roper Romps in many communities were the rules regarding the caftan. News accounts reiterated the importance of ease and comfort regarding Mrs. Roper’s costume. The caftan was supposed to be easy to find; some organizers even suggested stores where participants could be their own. Few, if any, specifications regarding caftans were issued publicly. It also did not have to be especially fancy, anything inexpensive you could get your hands on would suffice. Relatedly, the caftan or variation on Mrs. Roper’s costume should be comfortable. In fact, if participants wore a caftan, they *would* be comfortable. That, according to many accounts, was the point. Fun and joy were interlinked with comfort and ease.

She says Helen Roper had the right idea, when it came to idiosyncratic, loose-fitting fashion. ‘This is like pandemic attire,’ Pepperman says, smoothing her floor-length garment. ‘And now we have a license to be comfortable in our caftans in public. I think there might be a little post-pandemic retro thing going on right here’ (Article 104).

To have fun, participants should be comfortable, and to be present at the Romps, participants need the costume to be easy to find. Wigs allowed participants to forego concern about their hairstyles. These elements undergird the feeling rules of joy and fun.

Along with the caftan, the body was revealed to be significant to many Romps. Rompers were encouraged to move their bodies during organized activities (walking, dancing, singing, etc.). The form of the group activities varied, although they were often centered around a pub crawl or tour of local businesses. Organizers have incorporated other activities such as trivia, best-dressed competitions, karaoke, games of limbo, and charity drives. The physical movement expected at Roper Romps reinforces the body’s role in eliciting emotion (e.g., Johnston, 2021). Pub crawls, party games, wearing a costume—each of these requires attention to and movement of the physical body. Thus, to achieve the desire feeling, the body could be a way to engage in deep acting (Hochschild, 1979, 1983). Like Masi de Casanova et al. (2021, p. 813) in their qualitative examination of cosplayers, “embodying characters can create pleasurable feelings of empowerment or liberation.” This allows Rompers to manifest emotion through movement and physical adornment. Unlike those who seek exercise to elicit emotion (Alonso-Arbiol et al., 2024; Fullagar, 2008; Desjardins and Ketterling, 2023), the physical activity is integrated into the Romps; participants may not realize the effect of this movement, nor that of the costume and accessories, or their alignment with the feeling rules. By engaging in such leisure activities, they not only move their bodies, but also test out potential future identities (Ibarra and Petriglieri, 2010). This goes against the typical normative expectations regarding women’s physical movement in public. Woman can exercise in public spaces, but moving in physical ways dressed as a fictional television character violates folkways which are normalized by participation in an organized group.

Many Roper Romps accounts, especially in larger cities, promoted themselves as accepting of many identities, particularly those who identified as LGBTQ. This is likely connected to the original storyline of *Three’s Company*. First airing in 1976, *Three’s Company* began as a sitcom about three tenants, Jack, Janet, and Chrissy. In the pilot’s opening premise, the three characters claimed Jack was gay to legitimize their co-ed living situation in the eyes of the apartment’s owners, the Ropers. The promotion of many Romps then follows the character’s representation of acceptance, inviting anyone to participate. As stated in one article, “[b]y 2023 standards, the show might be considered retrograde in its attitudes toward feminism and homosexuality. But when it aired, its randy suggestiveness pushed broadcast boundaries. Mrs. Roper was its progressive Pole Star: Freethinking and voluptuary, she pooh-poohed her husband’s anti-gay slights and illustrated for Janet and Chrissy how an older woman could have sexual agency” (Article 82). Past participants’ accounts indicate a positive experience and a feeling of joy for all who attend. The discussion of Mrs. Roper’s personality and challenging of norms dictating sexuality were a frequent point of discussion by Rompers from previous events. Again, this indicates a joy that is promised through one’s participation. To feel joy, you should experience freedom, which is associated with the acceptance of all communities and the rejection of inequality.

Age was another lived experience addressed by attendees of previous Roper Romps. Participants were often middle-aged or older and found that by dressing like Mrs. Roper, they could channel the image Mrs. Roper created. By putting on a costume and embodying in her character (Hochschild, 1979), women could elicit a feeling of joy. Furthermore, women could embrace their age and push back on stereotypical ideas of older women. This was acceptable because Mrs. Roper herself did not follow those traditional rules, so when they participated in an event with her name, they did not either. This includes a rejection of, not traditional feeling rules, but the tools normative traditions offer to follow those rules. Smiling, laughing, and having fun were dictated by the events, but the bold abandonments of folkways influencing dress and behavior is a unique tool to satisfy those emotion requirements.

‘[A]s middle-aged women, we tend to—living in a society that sort of celebrates youth, I think we tend to feel like we’re fading into the background and this is really an opportunity to push against that and to feel lively and engaged and celebrated in one afternoon’ (Article 38).

Unlike Hybholt and Spotswood’s (2025) research, female participants cannot attend the Roper Romps routinely to gain pleasure from leisure; however, like their study results Rompers can leverage the event to “switch off” any emotions they want to avoid. As women shared their experiences, it becomes clear that the normative feeling rules of Romps allowed participants to shed the strictures of social convention and be proud of their age; the archetype insists that they do so. “That is, we’re not content to be invisible. Helen Roper was confident, fun, accepting, and very, very visible” (Article 23). Here, women are expected to be seen and publicly visible but also having fun.

### Gendered experiences and social construction of fun and community

Roper Romps also provide a window into the experiences of gender, race, and sexuality and how they shape what emotions are permitted in daily contexts. Though the data speaks explicitly to the effects of gendered feeling rules (Hochschild, 1983; Keys, 2010; Muñoz and Quirke, 2022; Andreasson et al., 2023; Olson, 2024) but also hint at the racialized and sexualized boundaries around emotions (Cottingham et al., 2017; Wingfield, 2010; Mai, 2022). The data reveal the desire for women to reclaim emotional agency through fun and community. The intensity of shared positive emotions is witnessed through descriptions of synchronized Romp activities and collective emotional arousal. The effects of past Romps indicate a collective effervescence (Durkheim, 1912/1995), social cohesion (Collins, 2004), and belonging that carries over into their mundane, everyday emotional experiences (Wettergren, 2009).

Romp participants are promised a joy that emerges from visibility, but Romps are also a means to escape. Women’s accounts of feeling free from the daily stresses of work and family were a recurring theme in relation to joy. The mention of Mrs. Roper’s husband and her ability to enjoy life indicate a possible nod to the gendered expectations and stresses of everyday life. One organizer-participant clearly articulates this:

“Bastan [a local organizer-participant] says the character of Mrs. Roper had a particular impact on the generation that grew up watching ‘Three’s Company,’ but that has continued into the modern day. In large part, she said that’s due to the stresses of working a stressful job. Bastan and most of her friends work in the nursing and hospice fields, while also raising a family. ‘Women in my age group, we’re kind of at this teeter where we’re from this era where we’re still traditional family,’ Bastan said. ‘We got married right out of college or right out of high school, we got a job. We had children early, and then when we got into our 40s we really didn’t know what we were supposed to do anymore. And so, Mrs. Roper is kind of like- it’s ok. You don’t have to know what to do anymore. You’re just going to live and you’re going to live each day one day at a time and enjoy it’ (Article 114).”

At the Romps, women should abandon those feelings and have fun. Recent historic events, like the COVID-19 pandemic have disproportionately affected women’s mental health (e.g., Qian and Fan, 2024). Community social events, such as Roper Romps, may help to alleviate these stressors. ‘It’s so fun getting out of your head and being OK with being absurd,’ she said. ‘It’s so freeing.” Quotes like this are emblematic of the concept of cognitive deep acting. When asked by a local reporter why Mrs. Roper Romp was so appealing, one participant responded, “Her joie de vive. She stands for confidence. I have never felt more young, thin, and pretty than I do today” (Article 88). Changing one’s thoughts or tapping into how Mrs. Roper would think allows participants to work through hesitation or discomfort and mentally take on the role of Mrs. Roper. By capitalizing on the gendered expectations of fun (Hautakangas, 2015), participants can use leisure to have the fun they may often miss.

Meeting others and sharing in the feelings of joy, socializing and participating in activities, pretending to be another person allows women to forget (albeit, temporarily) their ongoing challenges outside the Romp. The feeling rules established by these data iterate the expectation of joy for individuals, but importantly, the joy is tied to group participation. Emotions like happiness were regularly discussed in reference to others—laughing at themselves in front of others, laughing at others, having fun with friends, meeting new people, finding connections, coming together to share support, and participating in group activities.

The women showed up to the inaugural ‘downtown invasion’ after hearing about the Romp by word-of-mouth. ‘Most of us don’t know each other,’ Albritton said. Dell echoed Albritton’s observance. ‘These ladies were a lot of fun. I made some new friends,’ Dell said. The iconic Helen never met a stranger (Article 73).

By participating, Rompers are expected to reinforce not only the sense of community of which they are actively and physically a part, but there were also hints that Rompers should feel they are tied to a larger imagined community through participation (Wettergren, 2009). While contributing to and sharing in group emotion. It appears that not only do the Romps assist in evoking emotions, but some unwanted emotions may also be suppressed. Unlike Hochschild’s ethnographic research (1983), these women are not managing emotion on the job; yet they may engage in similar techniques of emotion management. Attending the Romps and engaging in playful and distracting activities suppress the stress and anxiety of recent events.

The past few years have been hard on her—‘Covid stuff, health stuff, family stuff,’ she explained. The Mrs. Roper Romp was a comfort, she said, because it allowed her and others to hit the pause button on ‘being caregivers and caretakers who need care ourselves. Please God, give me a frilly cocktail and a laugh with my friends,’ she said, ‘and just let it be easy for a minute’ (Article 82).

The depictions of shared emotion at Roper Romps resembled Durkheim’s (1912/1995) concept of collective effervescence; as attendees gathered, emotions were heightened by their shared co-presence, further imbuing the gatherings with meaning and feeling. Roper Romps in smaller communities shared a greater sense of shared support among participants. The physical and social presence in their own communities likely further enhanced their sense of joy.

There’s a sense of community […] You walk into a place dressed like Helen, and people just smile. Would you normally meet these people? Maybe, maybe not. It’s a really fun group of people, and I think we’re all looking for more connection in our lives’ (Article 76).

Because news articles promoted many smaller local Romps, and many Romps supported local charities over the course of the event, Rompers should leave feeling connected and supported by local residents and community members. As argued by Fine and Corte (2017), the physical and social space in which one operates can affect the kind of fun people may experience and allow them to feel more connected to their local communities (Simmel, 1984) and also reinforce local order and meaning (Mead, 1934).

### News stories and the dissemination of feeling rules

The journalistic accounts of previous Romps provide situational standards for the experience and expression of emotion (Brummer, 2024; Hochschild, 1983). Participants described how people align felt emotion with expected emotion through surface and deep acting (Hochschild,1979, 1983), cognitively reframing by using nostalgia (Smith and Kleinman, 1989) or justification through escape (Scott and Lyman, 1968). By sharing these accounts and disseminating these experiences, news stories create a sequence of narratives that establish a standard, legitimized emotional and normative description of what participants at Roper Romps can expect to experience. Normative expectations are prescriptive; there are descriptions of the location, what to wear, what to bring, and what activities will be made available, organizers often made the schedule clear to participants. However, there were few proscriptive norms. Fun and community were the primary outcome indicated by the news promotion. The situation is initially defined by the organizers, creating the social context in which participants will operate. Roper Romps, though novel and public, have their own social contexts; they are shaped, in part, by the organizers who promote these events, and past participants who share their experiences. These accounts contribute to the normative structure of future Romps, laying the groundwork for how participants should feel and how they should express their emotions. As evidenced by the following quote:

“[j]ust yourself, be yourself. You could be as in character as you want. Come on out. If you….if you do not feel like you are really Mrs. Roper, maybe you are a Mr. Roper, or maybe you are Janet or Jack or Chrissy. So we are gonna not…just going to tell you, no, you cannot come…come dressed however you like. Just come on out. Have a good time, and that’s what we are all here for (Article 31).”

Within the normative structure, the feeling rules for Roper Romps emerge. Like Hochschild’s (1983) ethnographic description of the feeling rules created for the Delta flight attendants, the feeling rules can be observed within the organizers’ explanations of the events, which vary across the nation as each individual Romps is constructed as its own situational context. The emotions which Rompers should experience come from both organizers and the participants from past Romps. By describing their past experiences, the participants indicate what emotions should be experienced and how to express those emotions. Here is a brief quote from one participant’s account: “‘It’s different and fun,’ She said it shows how you can have a good time without adhering to conventional norms, thanks due to the joy that comes from embracing spontaneity” (Article 92).

Just as feeling rules are dictated by promotion, so are display rules (Hochschild, 1979, 1983). Laughing, smiling, and embodying fun were common descriptions provided by reporters and participants. Thus, the promotion focuses not just on having fun but *appearing* as if having fun. “‘Hi Helen!’ one Helen said to a crowd of Helens. ‘Hi Helen!!!’ they shouted back. ‘That’s what’s awesome about it,’ the Helen said. ‘Instant community!” (Article 88). The greeting, “Hi, Helen” may also act as a rule reminder (Hochschild, 1983, p. 57) for fellow participants. This salute is a hint that the Rompers should remain in character, fun-loving, and friendly with others in the group. To be sure, the news stories indicate that Rompers should be aware that they are on stage to some degree, much like Goffman’s (1959) theory of the presentation of self. While in costume, the Rompers are front stage and should continue to show their joy and happiness. If some do not, they can be reminded of the performance. Some amount of surface acting (Hochschild, 1979) may be needed for those whose sense of joy may wane over the course of the day.

The central element in the transmission of the feeling rules is the character of Mrs. Roper. In *Three’s Company*, Mrs. Roper’s character focused on expressing her own sexual desires toward her (uninterested) husband. Her character exuded confidence, sexuality, and open-mindedness. The landlady’s personification of progressive values continues to align with modern views of gender and sexuality, appealing to many. “Mrs. Roper is a symbol of female cool and being exactly who you are” (Article 109). The social construction of this character as a cultural icon offers a symbolic prototype for the participants to model, physically and emotionally. She offers an unusual, if not mildly deviant, persona for participants to embody. This embodiment is one that allows participants to violate folkways in a way that is appropriately inappropriate. On the show, Mrs. Roper was “kooky” and represented a modern sentiment woven throughout the series plotlines. Her character provides participants today a role that not only gives them “permission” to be joyful, but the persona also expects them to engage in behaviors that elicit joy. As summed up in this comment: “‘She always fought for what she needed out of her life…She’s kind of this cool chick who enjoys life, and isn’t afraid to talk back to her husband, which was kind of unheard of back then’” (Article 113). The feeling rules demand that Rompers personify Mrs. Roper by embracing these collective definitions of identity and having fun.

## Discussion and conclusion

It is through social exchange that feeling rules and emotion management emerge (Hochschild, 1979, 1983). News media facilitate the emotional and normative expectations that participants at Roper Romps should find joy. No matter the length of the articles, the information provided contributed to the expectations for this event. Past participants shared accounts offering the clearest insight into what Rompers should and will feel, offering details about what brought them to a Romp and in what ways they were able to align their emotions with the feeling rules. News reporting is not the only approach to documenting the construction of feeling rules; however, it presents a unique data set to observe and explore a novel situational context. Furthermore, this technique may be especially useful for emerging pop culture trends. Though more detailed data sets are needed in future studies, journalistic sources can provide a starting point in which to situate these phenomena as they surface across communities.

Roper Romps are sites that legitimize women’s social connection and their public displays of joy. What may seem inappropriate (dressing in costume, having fun), is expected if not required within this specific situational context. Additionally, Roper Romps can also be utilized as a tool to experience the joy that they may struggle to evoke in everyday life. Though participants go about it in an outlandish and public way, women can express the joy that they are often expected to emote but managing it within a unique context. Women are often expected to display positive emotions. At a Romp they can, but through a method that might be more pleasurable and more authentic. Though Romps may look completely unlike themselves in appearance and behavior, they may actually feel more “like themselves.” Acting as a fictional character allows participants to evoke ideas of who they want to be and how they want to feel.

Articles not only shared the emotional experiences of the participants at the Romps, but the stories also primed future participants for the expected emotions. Reading anecdotes of past experiences and viewing promotional photographs and news videos of activities established the feelings that participants should experience if they attended. Joy and nostalgia are feelings that participants were expected to experience. For some women, the feeling rules and create a space for emotion management; participants likely attend because the goal is to have fun and can use the Romps as a device for evoking these emotions. If participants wore a caftan, put on a wig, and chunky jewelry, then they would feel happiness. Meeting with others, participating in activities, and supporting local businesses and organizations would produce joy. If they are unable to evoke these feelings in themselves, then they could look at the joy they bring to others (participants and bystanders, respectively) and call forth happiness of their own.

The Roper Romps also highlighted the pervasive gender inequalities that exist within many American families and within daily life more broadly. Many participants described the stresses of managing and caring for family members. Several explicitly connected their experiences to the COVID-19 pandemic. These experiences are supported by recent literature (e.g., Qian and Fan, 2024). Furthermore, many of the participants fell within the age range of the sandwich generation. As a “woman in the middle” (Schlesinger and Raphael 1993), participants may be actively caring for aging parents or older relatives while also parenting their own children. Events like these allowed participants to shed the stresses of everyday life and become someone else for a few hours (i.e., Masi de Casanova et al., 2021). And not just anyone—a female character known for her authenticity and confidence, who also wore comfortable clothing. Like Bronies (Hautakangas, 2015), Roper Romp attendees, particularly women, leverage the gendered expectations of women. At Roper Romps, women could be loud public spectacles, dressing flamboyantly but comfortably, and could experience (and express) unabashed pleasure, stretching the social rules about women’s behavior, self-expression, and personal feelings.

Mrs. Roper’s character was viewed as outside the norm, if not inappropriate in some ways; therefore, the behavior encouraged at a Romp is considered normative given the context. Because the events are still new to many across the country, in the spirit of Mrs. Roper, part of the pleasure they derive comes from the inappropriateness of the event itself. To be sure, if everyone is there to have fun and the fun it situated around a fictional female character, women may feel safer and more willing to express and feel joy compared to other situational contexts. Acceptance of all identities creates a (potentially) safe space for women, LGBTQ communities, and people of color. The promised inclusion, the novelty of a pop culture phenomenon, and its “appropriate” violation of cultural folkways are likely attractive those who are looking to have a good time—women in particular.

There are several limitations to these findings. First, there were only 112 news articles included in the database between 2023 and 2024. In addition, unlike previous research (e.g., Masi de Casanova et al., 2021) many stories shared limited information on the upcoming Romp and no anecdotal details. Future qualitative studies should construct ethnographic examinations and in-depth interviews (Hochschild, 1983) of Romps to form a more grounded understanding of these events. Further scholarship could more clearly delineate the intersection of leisure and emotion management.

Second, one of the motivations behind the news reporting for local Roper Romps was promotion. Thus, the highlighted stories and personal experiences would likely be positive. Local organizers focused on the fun and freedom offered at the Roper Romps. While the authenticity of the accounts is not necessarily in question, the scope of these data is constrained by the purpose of the reporting. When community organizers are seeking attendees, they tend to highlight the positive aspects of the event. Thus, it follows that participants would expect an event to be fun and create joy. Cosplayers are a comparable group; they construct a pleasurable experience when attending conventions. It’s partly why they do it—because it’s fun (Masi de Casanova et al., 2021). Thus, scholars should continue to explore emotions and emotion work at Roper Romps, as well as how social factors such as gender, race, and age shape these processes.

Finally, because of the vast array of communities in which Romps are held and the differential levels of commitment expressed, the edict of inclusion was not unilaterally fulfilled. People of all races and ethnicities are accepted by many Romps, but this may not translate into many smaller communities or groups. Though the promotion of Roper Romps indicates that everyone should be happy and that normatively, everyone will be included. There is no guarantee that all members will enjoy the Romp, nor will they feel welcome. One article highlighted a participant’s explicit desire for representation. “‘In the TikTok videos [of the Romps], I didn’t see any African American Helens,’ she explains. ‘So I said to my cousins, we’re just going to go out there and represent. I don’t know if we’ll be the only three melanin people there, but we’re going to have fun’” (Article 104). Though little detail is shared in this quote, it does indicate some amount of potential emotion management beyond what white participants may engage. Like Mai’s (2022) study on Vietnamese LGBT activists, there may be a mismatch between what is seen front stage versus backstage.

This paper contributes to existing sociological literature in several ways. First, the author brings a previously unexamined situational context which further articulates the relevance and strength of Hochschild’s (1979, 1983) theories. Pop culture phenomena, like Roper Romps, continue to offer sociologists fruitful opportunities to apply and extend theoretical concepts. The application of emotion management techniques and feeling rules allows us to more fully grasp the intricacies of and connection to emotion and leisure. More specifically, the ways in which people elicit and release unwanted emotions (joy and stress, respectively). Further, the author observes how participation in a group facilitates this experience. This is especially needed when the events are seemingly guided by gendered feeling rules. How groups and news media construct these emotional expectations is useful to our understanding of a spectrum of situations. Further defining the cultural and contextual processes by which emotions are established and then evoked and suppressed.

## Data Availability

The raw data supporting the conclusions of this article will be made available by the authors, without undue reservation.
